# Polygenic liability for anxiety in association with comorbid anxiety in multiple sclerosis

**DOI:** 10.1002/acn3.52025

**Published:** 2024-05-07

**Authors:** Kaarina Kowalec, Arvid Harder, Casandra Dolovich, Kathryn C. Fitzgerald, Amber Salter, Yi Lu, Charles N. Bernstein, James M. Bolton, Gary Cutter, John D. Fisk, Joel Gelernter, Lesley A. Graff, Sara Hägg, Carol A. Hitchon, Daniel F. Levey, Fred D. Lublin, Kyla A. McKay, Scott Patten, Amit Patki, Murray B. Stein, Hemant K. Tiwari, Jerry S. Wolinsky, Ruth A. Marrie

**Affiliations:** ^1^ Rady Faculty of Health Sciences University of Manitoba Winnipeg Canada; ^2^ Department of Medical Epidemiology & Biostatistics Karolinska Institutet Solna Sweden; ^3^ Department of Internal Medicine Max Rady College of Medicine, Rady Faculty of Health Sciences, University of Manitoba Winnipeg Canada; ^4^ Department of Neurology Johns Hopkins School of Medicine Baltimore Maryland USA; ^5^ Department of Neurology UT Southwestern Dallas Texas USA; ^6^ Department of Psychiatry Max Rady College of Medicine, Rady Faculty of Health Sciences, University of Manitoba Winnipeg Canada; ^7^ Department of Biostatistics University of Alabama at Birmingham Birmingham Alabama USA; ^8^ Nova Scotia Health and Departments of Psychiatry, Psychology & Neuroscience, and Medicine Dalhousie University Halifax Nova Scotia Canada; ^9^ Department of Psychiatry Yale University, School of Medicine New Haven Connecticut USA; ^10^ Department of Psychiatry VA Connecticut Healthcare System New Haven Connecticut USA; ^11^ Department of Clinical Health Psychology Max Rady College of Medicine, Rady Faculty of Health Sciences, University of Manitoba Winnipeg Canada; ^12^ Department of Rheumatology Max Rady College of Medicine, Rady Faculty of Health Sciences, University of Manitoba Winnipeg Canada; ^13^ Icahn School of Medicine at Mount Sinai New York New York USA; ^14^ Department of Clinical Neuroscience Karolinska Institutet Solna Sweden; ^15^ Department of Community Health Sciences Cumming School of Medicine, University of Calgary Calgary Canada; ^16^ Department of Psychiatry University of California San Diego La Jolla California USA; ^17^ Department of Neurology McGovern Medical School, The University of Texas Health Science Center at Houston (UTHealth) Houston Texas USA; ^18^ Department of Community Health Sciences Max Rady College of Medicine, Rady Faculty of Health Sciences, University of Manitoba Winnipeg Canada

## Abstract

**Objective:**

Comorbid anxiety occurs often in MS and is associated with disability progression. Polygenic scores offer a possible means of anxiety risk prediction but often have not been validated outside the original discovery population. We aimed to investigate the association between the Generalized Anxiety Disorder 2‐item scale polygenic score with anxiety in MS.

**Methods:**

Using a case–control design, participants from Canadian, UK Biobank, and United States cohorts were grouped into cases (MS/comorbid anxiety) or controls (MS/no anxiety, anxiety/no immune disease or healthy). We used multiple anxiety measures: current symptoms, lifetime interview‐diagnosed, and lifetime self‐report physician‐diagnosed. The polygenic score was computed for current anxiety symptoms using summary statistics from a previous genome‐wide association study and was tested using regression.

**Results:**

A total of 71,343 individuals of European genetic ancestry were used: Canada (*n* = 334; 212 MS), UK Biobank (*n* = 70,431; 1,390 MS), and the USA (*n* = 578 MS). Meta‐analyses identified that in MS, each 1‐SD increase in the polygenic score was associated with ~50% increased odds of comorbid moderate anxious symptoms compared to those with less than moderate anxious symptoms (OR: 1.47, 95% CI: 1.09–1.99). We found a similar direction of effects in the other measures. MS had a similar anxiety genetic burden compared to people with anxiety as the index disease.

**Interpretation:**

Higher genetic burden for anxiety was associated with significantly increased odds of moderate anxious symptoms in MS of European genetic ancestry which did not differ from those with anxiety and no comorbid immune disease. This study suggests a genetic basis for anxiety in MS.

## Introduction

Anxiety disorders are an important cause of disability worldwide,^1^ and together with depressive disorders cost approximately $90 billion annually in personal health spending in the United States alone.^2^ Comorbid anxiety disorders occur more often in persons with multiple sclerosis (PwMS) than in the general population (incidence rate ratio: 1.47, 95% CI: 1.34–1.61),^3^ and are associated with greater annual disability progression,^4^ and cognitive dysfunction.^5^ Elevated anxiety symptoms are also common in PwMS^6^, ^7^ and are associated with work and cognitive impairment.^8^ In PwMS, in general, anxiety is underdiagnosed and undertreated.^9^ Thus, improved identification of PwMS with a high risk for comorbid anxiety would be desirable to facilitate screening, earlier diagnosis, and intervention.

The etiology underlying MS and comorbid anxiety is unknown but is thought to be related to genetics and environment based on studies within the general population or because of MS‐related pathology or a combination of these. Factors associated with anxiety disorders in PwMS include female sex,^10^ having comorbid depression,^10^ lower education,^11^ and smoking.^11^ Anxiety occurs prior to MS symptoms or following a diagnosis,^12^ highlighting the need to study anxiety in MS regardless of the timing of onset of the anxiety disorder. Whether genetic variation affects the risk of anxiety in PwMS differently than in individuals without MS is unknown. In the general population, the single nucleotide polymorphism (SNP)‐based heritability of anxiety, as measured either as a continuous or categorical anxiety phenotype, ranges from 5.6% to 9.5%.^13^, ^14^ Anxiety is a polygenic disorder,^13^ and previous studies in MS have investigated only a limited number of genetic variants in association with anxiety in PwMS. One candidate gene study found an association between pro‐inflammatory cytokine (IL‐1β) genetic variation and anxiety in both PwMS and in healthy controls, highlighting a role for immune factors in the development of anxiety, but this was not specific to MS.^15^ A Mendelian randomization study did not find any causal relationship between MS and anxiety, although only five instrumental variables for anxiety were used in the analyses.^16^

Polygenicity, as measured by a polygenic score (PGS), is the cumulative genetic burden as indexed by the number of common genetic variants associated with a trait, weighted by their effect sizes from previous genome‐wide association studies. Genetic variants included in the PGS are largely determined at birth making them a useful exposure to investigate in an association with an outcome as it can exclude reverse causation as an explanation for the association. Recent genome‐wide analyses of current anxiety symptoms as measured by the Generalized Anxiety Disorder 2‐item scale (GAD‐2) included 199,611 individuals of European genetic ancestry from the US‐based Million Veterans Program and identified five genome‐wide significant loci (*P* < 5 × 10^−8^).^13^ While this study identified common genetic variation associated with anxiety symptoms in a large population, the findings may not translate to PwMS for a number of reasons including that they did not assess comorbid immune diseases nor the female preponderance noted with MS. An association between the GAD‐2 PGS and anxiety has not been shown before in the MS population and would be important to evaluate the postulated association. Thus, we aimed to examine the association between the GAD‐2 PGS and the presence of anxiety specifically in PwMS. We performed site‐specific analyses and then meta‐analyzed data from individuals from Canada, the United Kingdom, and the United States. We hypothesized that higher GAD‐2 PGS would be associated with a greater likelihood of comorbid anxiety in PwMS compared to those without anxiety, as defined by multiple measures of anxiety.

## Methods

### Study design and samples

We applied a case–control study design using three existing cohorts from Canada, the United Kingdom, and the United States. Further details can be found in Data S1.

#### Canada (IMID study)

A prospective 3‐year, longitudinal study of persons with immune‐mediated inflammatory diseases (IMID) was utilized.^17^ Briefly, participants residing in Manitoba, Canada were recruited between November 2014 and July 2016. Multiple recruitment methods were used such as advertisements placed in private medical clinics, hospitals, and educational institutions. Participants had to be sufficiently proficient in English to complete study questionnaires and were ≥18 years old. In this study, we included three groups of participants: (1) PwMS, (2) a lifetime history of anxiety disorders but no immune disease, and (3) healthy controls.

#### United Kingdom (UK Biobank)

The UK Biobank (UKB) is a population‐based cohort of ~500,000 individuals aged 37–73 years from the UK, recruited from 2006 to 2010.^18^ At an assessment center, participants answered touchscreen questionnaires about diseases, which was then followed by a research nurse‐led interview for further details regarding the diseases reported. Linkage to hospital records was also included to capture ICD‐10 diagnoses, with a subset also completing a mental health questionnaire. From this cohort, we selected the same three groups of participants as the Canada study.

#### USA (CombiRx trial)

The CombiRx trial was a randomized, multi‐center, Phase III clinical trial of combination MS disease‐modifying therapies (interferon‐β1a and glatiramer acetate vs. either agent alone; ClinicalTrial.gov: NCT00211887 funded by the National Institute of Neurologic Disorders and Stroke [phase III study: UO1NS045719]).^19^ Completion of the 3‐year core study was then extended to include an additional 4 years (maximum of 7 years total follow‐up). As this was an MS clinical trial, only PwMS were included who were naïve to disease‐modifying therapy at entry, with and without a medical history of anxiety.

### Participant measures and definitions

Our outcome was comorbid anxiety. From the Canada and UKB participants, we defined one case group (PwMS and anxiety) and three control groups: (1) PwMS and no anxiety, (2) anxiety and no immune disease, and (3) healthy. For excluding immune diseases in Group 2, we excluded those with inflammatory bowel disease, MS, rheumatoid arthritis, celiac disease, psoriasis, and Sjögren's syndrome.^17^ In the USA cohort, which was an MS clinical trial, only PwMS and anxiety (cases) and PwMS and no anxiety (control) could be used. Our outcome was comorbid anxiety. Anxiety was assessed after the MS diagnosis by multiple measures: current symptoms, lifetime interview‐diagnosed, and lifetime self‐report physician‐diagnosed.

#### Canada

The following were details captured at the baseline visit using a self‐reported questionnaire: date of birth, sex, annual household income (Canadian dollars: <$50,000, ≥$50,000, or “decline to answer”), physician‐diagnosed comorbidities, highest level of education attained (high school or lower which includes elementary school, junior high school, high school diploma/GED vs. above high school: college, technical/trade, university vs. other) and years of education, and smoking history (smoked ≥100 cigarettes in their lifetime).^17^ Weight and height were measured by a research assistant to compute BMI (kg/m^2^).

PwMS had neurologist‐confirmed MS. Anxiety was defined four ways: (1) current symptoms: the GAD‐7, a validated 7‐item self‐reported tool that assesses anxiety symptom severity over the previous 2 weeks and employs a validated cutoff for the presence of at least moderate anxiety symptoms (GAD‐7 ≥ 10),^20^ (2) current symptoms: employing the first two questions of the GAD‐7, referred to as the GAD‐2, to align with that of the genome‐wide association study of GAD‐2,^13^ (3) lifetime anxiety disorders: assessed using the gold standard structured clinical interview for diagnostic and statistical manual of mental disorders, version 5 (SCID‐DSM‐5),^21^ and (4) lifetime self‐report of anxiety disorder diagnosed by a physician, which was derived from a questionnaire validated for use in PwMS.^22^ The SCID‐DSM‐IV was used as this was the prevailing version at the time the Canadian study was designed;^21^ but obsessive‐compulsive disorder and posttraumatic stress disorder were then excluded to better reflect anxiety disorders defined using DSM‐5 (current diagnostic criteria).^23^ Anxiety was defined the same way in PwMS and in persons without an immune disease. Healthy controls did not have any chronic medical condition (Table S1), a known cognitive impairment, any positive response to the SCID‐DSM screening questions for depressive or anxiety disorders, any head injury associated with loss of consciousness or amnesia, or chronic medication use with the exceptions of contraceptives, hormone replacement therapy, transient antibiotic use, or multivitamins.

#### United Kingdom

Self‐report questionnaires captured similar information to that of the Canadian cohort, with further details in the Supplement. PwMS were defined using ≥2 of the following: primary or secondary hospital admission code (International Classification of Disease (ICD)‐version 10: G35), lifetime self‐reported condition (data‐field 20002: 1261) or lifetime self‐reported MS disease‐modifying therapy (any of interferon‐β/interferon‐β‐1b or 1a/avonex/betaferon, glatiramer/Copaxone).^24^ Newer MS disease‐modifying therapies were not included as the recruitment period predated their use.

Four definitions of anxiety were like that of the Canadian cohort, including (1) GAD‐7 ≥ 10, (2) GAD‐2 score, (3) self‐reported lifetime anxiety, (4) lifetime anxiety diagnosis using a combination of the presence of primary or secondary hospital admission codes for anxiety (ICD‐10: F40 or F41) or Composite International Diagnostic Interview‐short form (CIDI‐SF) based anxiety disorder, which is a fully structured interview.^25^ For healthy controls, we excluded those with chronic medical conditions using hospital admission ICD‐10 codes or self‐reported conditions (Table S1).

#### USA

Clinical and demographic details were collected at the enrolment visit for the CombiRx clinical trial, including sex, age, BMI, highest level of education attained (high school/more than high school), and smoking history (ever/never). This cohort was composed of neurologist‐confirmed PwMS. Self‐reported anxiety was assessed by querying medical history (at trial enrollment) during a clinician interview and the use of concomitant medications (indication was not collected) during enrollment and follow‐up.^26^ In this sample, there were no structured clinical interviews for psychiatric disorders, no linkage to hospital records, and no anxiety symptom measures.

### Genotyping, quality control, and PGS computation (exposure)

Genotype data were available for 409 Canadian, 599 USA, and 487,410 UK participants. This was after site‐specific quality control, and then imputation which is a standard practice in genetics to infer genetic markers that were not directly genotyped.^27^ Sample genotyping and processing are described in detail elsewhere for all samples^18^, ^28^, ^29^ and are summarized in Data S1. Using principal components analysis, we selected only those participants with European genetic ancestry to match with that of the individuals included in the GAD‐2 genome‐wide association study (Data S1).^13^ This decision was made to optimize PGS association and is supported by studies finding that including non‐European participants can result in reduced PGS association of diseases because of differences in allele frequencies, linkage disequilibrium, and causal effect sizes between populations.^30^, ^31^ Further to this, we had a limited number of MS participants of non‐European genetic ancestry (African: *N* = 11, South Asian *N* = 6, all other ancestries: *N* < 5). From all three cohorts, genotyped and imputed genetic variants were subject to site‐specific quality control. The exposure (i.e., PGS) was calculated using summary statistics from the recent genome‐wide association study of GAD‐2^13^ as the sum of the risk allele scores, weighted by the effect size (Data S1) using the genetics software PLINK (v1.90). We standardized all PGS to a mean of 0 (SD = 1) for ease of interpretation. For well‐powered PGS association analysis, it is recommended to include a minimum of 100 individuals in the target set and a PGS only if the trait has an SNP‐based heritability (*h* ^2^_snp_) >0.05,^32^ which for the GAD‐2 exceeds (*h* ^2^_snp_ = 0.0558^13^).

### Statistical analyses

Characteristics of the three cohorts (Canada, UKB, USA) were described using median (IQR), mean (SD), or frequency (%).

We tested whether the GAD‐2 PGS (exposure) was associated with four anxiety phenotypes (outcomes) in Canada and UKB and one outcome in the USA cohort. The outcome was any of three categorical measures (yes/no): current GAD‐7 ≥ 10, lifetime DSM‐5 anxiety disorders, or lifetime self‐reported physician‐diagnosed anxiety, and one outcome was treated as a continuous variable (current GAD‐2 score). We used multivariable logistic regression (binary outcome) or linear regression (continuous measure) and reported the results as odds ratios (95% confidence intervals, 95% CI) or beta estimates (standard error) per 1‐SD increase in PGS. We included the following covariates in all models: age at baseline (continuous), sex, and the first five genetic ancestry principal components, with the addition of a disease group indicator variable when the outcome was continuous.

When the outcome was categorical, we compared PwMS‐comorbid anxiety (cases) to (1) PwMS/no comorbid anxiety (primary analyses), (2) anxiety/no comorbid immune disease, and (3) healthy controls. When the outcome was continuous (i.e., GAD‐2 score), all participants were included, with a disease group indicator variable. The results for each comparison were performed by cohort and then meta‐analyzed across samples using a fixed‐effect inverse variance‐weighted model, when between study heterogeneity^33^ [*I* ^2^] was ≤50% or random‐effect inverse variance‐weighted model when *I* ^2^ > 50%.^34^ Last, as the relative effect of MS on the increasing incidence of anxiety is greater in men than women,^35^ we performed exploratory sex‐stratified analyses comparing PwMS/anxiety (case) to PwMS/no anxiety (control), with the inclusion of a sex × GAD‐2 PGS interaction term in the unstratified analyses.

Statistical significance level was set at *P* ≤ 0.05. Missing data were limited and were not imputed (other than the genetic data). Cell sizes <5 were suppressed. Analyses were performed using *R* for Statistical Computing (v.4.1.2)^36^ with the following packages: *tidyverse*, *cowplot* (figures), and *metafor*.

### Standard protocol approvals, registrations, and patient consent

All participants provided informed consent. The relevant site provided ethical approval for this study (University of Manitoba Health Research Ethics Board and Shared Health/Winnipeg Regional Health Authority; CombiRx [USA] respective collecting institutions; and Karolinska Institutet Ethics Committee for access to the UKBB data). This research has been conducted using the UK Biobank Resource under Application Number “22224.”

## Results

A total of 71,343 European genetic ancestry participants from Canada (*N* = 334; 212 MS), UKB (*N* = 70,431; 1,390 MS), and USA (*N* = 578 MS) were included in the study (Table 1). In the USA cohort, PwMS were younger at study enrolment than those in the Canadian and UKB cohorts. As a population‐based cohort with a known “healthy volunteer” bias, the UKB had lower rates of self‐reported anxiety in PwMS (4.7%), compared with the Canadian (21.2%) and USA samples (20.5%), which were collected from MS clinics and clinical trials. Across all three sites, 229 (10.5%) PwMS self‐reported lifetime anxiety, 101 (4.6%) PwMS met the criteria for a lifetime DSM‐5 anxiety disorder, and 70 (3.2%) PwMS reported current moderate anxious symptoms (GAD‐7 ≥ 10, Table S3). The overlap between the three categorical anxiety measures varied (Figure S1), but a similar proportion of Canadian (10.9%) and UKB (9.2%) participants with MS had comorbid anxiety or elevated anxiety symptoms as defined by all three categorical measures.

**Table 1:** Participant characteristics.

	Canada, *n* = 334				UKB, *n* = 70,431				USA, *n* = 578	
	MS, anxiety	MS, no anxiety	Anxiety, no immune disease	Healthy	MS, anxiety	MS, no anxiety	Anxiety no immune disease	Healthy	MS, anxiety	MS, no anxiety
*N*	45	167	76	46	65	1325	14,820	54,221	119	459
Females^1^	41 (91.1)	132 (79.0)	65 (85.5)	29 (63.0)	52 (80)	963 (72.7)	9840 (66.4)	27,954 (51.6)	93 (78.2)	324 (70.6)
Age, years^2^	47.4 (13.7)	52.5 (12.1)	43.4 (13.4)	43.9 (18.0)	53.1 (7.5)	55.5 (7.5)	55.2 (7.6)	52.6 (7.9)	37.3 (9.3)	38.8 (9.4)
Highest education^1^										
≤High school	15 (33.3)	56 (33.5)	20 (26.3)	12 (26.1)	28 (43.1)	545 (41.3)	5766 (38.9)	21,888 (40.4)	24 (20.2)	62 (13.5)
>High school	30 (66.7)	105 (62.9)	53 (69.7)	32 (69.6)	35 (53.8)	559 (42.2)	8039 (54.2)	26,831 (49.4)	83 (69.7)	337 (73.4)
Other	0	6 (3.6)	3 (3.9)	2 (4.4)	2 (3.1)	201 (15.2)	923 (6.3)	5,078 (9.4)	0	0
Declined	0	0	0	0	0	20 (1.5)	92 (0.6)	424 (0.8)	12 (10.1)	60 (13.1)
Education, years^2^	14.4 (3.1)	14.1 (2.5)	15.2 (2.9)	16.3 (2.9)	15.5 (4.7)	13.9 (5.0)	15.4 (4.7)	14.8 (4.9)	N/A	N/A
Income^1^										
<$50,000 CAD	18 (40)	50 (29.9)	29 (38.2)	12 (26.1)	25 (39.1)	629 (51.4)	5641 (39.1)	15,797 (29.9)	N/A	N/A
≥$50,000 CAD	23 (51.1)	97 (58.1)	43 (56.6)	32 (69.6)	36 (56.2)	469 (38.5)	7824 (54.4)	32,209 (61.2)	N/A	N/A
Declined	4 (8.9)	20 (12)	4 (5.3)	2 (4.4)	3 (4.7)	120 (9.9)	928 (6.4)	6,215 (11.5)	N/A	N/A
Ever smoker^1^	27 (60)	96 (57.5)	39 (51.3)	8 (17.4)	33 (50.8)	702 (53.0)	6,940 (46.8)	20,470 (37.8)	64 (12.6)	191 (33.0)
BMI (kg/m^2^)^2^	29.9 (7.7)	28.4 (7.2)	28.0 (6.5)	24.9 (3.5)	26.2 (5.1)	26.3 (5.1)	26.8 (4.8)	26.0 (3.9)	27.8 (6.0)	28.9 (6.7)
Overweight^1^	15 (33.3)	57 (34.1)	22 (28.9)	16 (34.8)	22 (33.8)	440 (33.2)	5871 (39.6)	22,828 (42.1)	40 (33.6)	142 (30.9)
Obese^1^	18 (40.0)	48 (28.7)	27 (35.5)	6 (13.0)	13 (20)	256 (19.3)	3075 (20.7)	7,594 (14.1)	36 (30.3)	169 (36.8)
DSM‐5 lifetime anxiety disorder^1^	27 (60)	27 (16.2)	70 (92.1)	0	0	13 (0.98)	242 (1.6)	0	N/A	N/A
Self‐reported anxiety^1^	45 (100)	0	76 (100)	0	65 (100)	0	14,820 (100)	0	119 (100)	0
GAD‐2 score^2^	2.6 (1.9)	1.5 (1.6)	4.0 (1.6)	0.3 (0.7)	1.8 (1.9)	0.7 (1.23)	1.41 (1.7)	0.30 (0.7)	N/A	N/A
GAD‐7 score^2^	7.3 (5.5)	4.0 (4.4)	12.1 (5.5)	0.7 (1.5)	5.6 (5.7)	2.4 (3.5)	4.35 (4.8)	1.01 (1.8)	N/A	N/A
GAD‐7 ≥ 10^1^	17 (37.8)	27 (16.2)	52 (68.4)	0	11 (16.9)	15 (1.13)	1991 (13.4)	62 (0.1)	N/A	N/A
GAD‐2 PGS^2^	−0.02 (0.1)	−0.1 (0.1)	0.27 (0.9)	−0.14 (0.6)	−0.04 (1.0)	0.02 (0.9)	0.03 (1.0)	−0.04 (1.0)	0.04 (1.1)	−0.01 (1.0)

Anxiety as a participant category is defined using self‐reported anxiety for all samples as this was the measure available across all three cohorts. Missing *N*: Canada BMI: *N* = 6, USA ever smoker: *N* = 72, UKB BMI: *N* = 351, UKB ever smoker: *N* = 320. BMI, body mass index; PGS, Polygenic score.

^1^ Categorical variable reported as *n* (%).

^2^ Continuous variable reported as mean (SD).

We assessed whether there was an association between the GAD‐2 PGS and the three categorical measures and one continuous measure of lifetime or current anxiety. Our primary analyses included a comparison between PwMS/anxiety to PwMS/no comorbid anxiety. Our multivariable regression meta‐analyses of the two cohorts with GAD‐7 scores (Canada, UKB) identified that in European genetic ancestry PwMS, each 1‐standard deviation (SD) increase in the GAD‐2 PGS was associated with a ~50% increased odds of having comorbid moderate anxiety symptoms (GAD‐7 ≥ 10) compared to those with less than moderate symptoms among PwMS (OR per 1‐SD increase in GAD‐2 PGS: 1.47, 95% CI: 1.09–1.99, Table 2). When we examined either lifetime DSM‐5 anxiety disorder or self‐reported physician‐diagnosed anxiety as the categorical outcomes, the multivariable regression effects were in a similar direction to that of current moderate anxiety symptoms, although they were not statistically significant (Table 2 and Table S3).

**Table 2:** Multivariable logistic regression analyses investigating the association between the GAD‐2 polygenic score and comorbid anxiety in multiple sclerosis compared to persons with multiple sclerosis/no anxiety.

Binary outcome	Sample			Meta‐analysis
	Canada	UK	USA	
Current GAD‐7 ≥ 10	1.24 (0.84–1.81); 0.9	**2.03 (1.2–3.4); 0.006**	N/A	**1.47 (1.09–1.99), 0.02, 58.2%** ^**1**^
Lifetime DSM‐5 anxiety disorders	1.35 (0.95–1.90); 0.08	1.03 (0.76–1.40); 0.9	N/A	1.16 (0.93–1.45), 0.18, 29.8%
Lifetime self‐reported physician diagnosed anxiety	1.17 (0.82–1.67); 0.1	0.93 (0.71–1.21); 0.7	1.20 (0.98–1.47), 0.08	1.07 (0.93–1.23), 0.35, 29.6%

Data represented as odds ratio (95% CI), *P*‐value, *I* ^2^ (for meta‐analyses). Bolded *P*‐value indicates *P* ≤ 0.05. The outcome is multiple sclerosis/anxiety (case) compared with multiple sclerosis/no anxiety (control). Each anxiety measure is assessed as a separate model and includes the polygenic score for GAD‐2, the first five genetic ancestry principal components, age, and sex.

^1^ Random‐effect inverse variance‐weighted model, whereas others used a fixed effect.

In our secondary analyses, comparing PwMS/anxiety to healthy controls, we found a 1‐SD GAD‐2 PGS increase was associated with similar odds of comorbid moderate anxiety to that of the primary (OR per 1‐SD increase in GAD‐2 PGS: 1.45, 95% CI: 1.05–1.98, Table S4). Last, when we compared the GAD‐2 PGS between anxiety occurring as a comorbidity in PwMS to that where anxiety was the index disease (i.e., anxiety without an immune disease) we did not observe a statistically significant difference (Table S4).

Stratifying the primary comparison of PwMS/anxiety compared to PwMS/no anxiety by sex yielded similar proportions of males and females in each sample by anxiety measure (Table S5). The sex‐stratified meta‐analyses for the current moderate anxiety symptoms (GAD‐7 ≥ 10) revealed a larger effect for males compared to females (OR = 3.89 for males vs. OR = 1.43 for females), but this effect was not significant in males. In addition, the formal tests for effect modification (sex × GAD‐2 PGS interaction term) were not significant (Table S6).

We also investigated the association between GAD‐2 PGS and the first two questions of the GAD‐7 scale (i.e., the GAD‐2) as a continuous score, including all possible samples in the Canadian and UK cohorts (*N* = 334 Canada, *N* = 45,195 UK). We found each 1‐SD increase in the GAD‐2 PGS was associated with higher GAD‐2 scores (β = 0.08, SE = 0.01, *P* < 0.001, Table 3). We included interaction terms for the GAD‐2 PGS by disease type and sex in both the Canadian and UK cohorts (Table 3 footnote) but did not find replication of any significant interaction in either cohort. However, a significant interaction for the GAD‐2 PGS in the anxiety/no immune disease participant group was detected in the UKB cohort (β = 0.07, *P* < 0.001). There was no difference upon stratifying these results by sex, with results in the same direction and magnitude as that of the full results (Table 3).

**Table 3:** Linear regression analyses investigating the association between the GAD‐2 polygenic score and higher anxiety symptoms (GAD‐2) as a continuous measure in the Canadian and UKB samples, including participants with MS, anxiety, and no immune disease and healthy controls.

	Canada			UKB			Meta‐analyses		
	All (*N* = 334)	Sex‐stratified		All (*N* = 45,195)	Sex‐stratified		All (*N* = 45,529)	Sex‐stratified	
		Female	Male		Female	Male		Female	Male
GAD‐2 PGS	0.15 (0.09); 0.09	0.12 (0.10); 0.25	0.27 (0.21); 0.27	0.08 (0.01), **<0.001**	0.09 (0.01), **<0.001**	0.07 (0.01), **<0.001**	0.08 (0.01), **<0.001,** 0%	0.09 (0.01), **<0.001,** 0%	0.07 (.01), **<0.001,** 0%
Disease group: multiple sclerosis	1.64 (0.26), **<0.001**	1.64 (0.32); **<0.001**	1.51 (0.47); **<0.001**	0.56 (0.07), **<0.001**	0.62 (0.01), **<0.001**	0.42 (0.14), **<0.01**	0.63 (0.07), **<0.001,** 94%^1^	0.62 (0.01), **<0.001,** 90.1%	0.51 (.13), **<0.001**, 79.3%
Disease group: anxiety	3.64 (0.29), **<0.001**	3.52 (0.36); **<0.001**	4.14 (0.59); **<0.001**	1.10 (0.02). **<0.001**	1.09 (0.02), **<0.001**	1.11 (0.02), **<0.001**	1.11 (0.02), **<0.001,** 98.7%^1^	1.09 (0.02), **<0.001**, 97%	1.11 (0.02), **<0.001**, 96.2%

The outcome in all models is the GAD‐2 as a continuous measure. The exposures include all factors listed in addition to the first five genetic ancestry principal components, age, and sex. Results are reported as beta estimate (SE), *P*‐value. Bolded *P*‐value indicates *P* ≤ 0.05. PGS, polygenic score.

^1^ Random‐effect inverse variance‐weighted model, whereas others used a fixed effect. For the disease group variable, the reference category is the healthy controls. We also included the following interaction terms to the full model in the Canada sample: GAD‐2 PGS × anxiety (β = −0.01, *P* = 0.9), GAD‐2 PGS × MS (β = −0.07, *P* = 0.7), GAD‐2 PGS × sex (β = 0.17, *P* = 0.4), and in UKB sample: GAD‐2 PGS × anxiety (β = 0.07, *P* =< **0.001**), GAD‐2 PGS × MS (β = 0.14, *P* = 0.06), GAD‐2 PGS × sex (β = 0.01, *P* = 0.5).

## Discussion

Using a large sample of >70,000 individuals from three countries and multiple control groups, we confirmed our hypothesis that the GAD‐2 PGS is associated with comorbid anxiety in MS. A 1‐SD increase in the GAD‐2 PGS was associated with ~50% increased odds for moderate anxious symptoms in PwMS compared to those with less than moderate anxiety symptoms. Results in the same direction, albeit with smaller effects, were noted for the categorical measures of lifetime anxiety. We also found that the association of GAD‐2 PGS did not differ when comparing anxiety among PwMS to individuals without an immune disease. Thus, we had no data to reject the hypothesis that based on our current knowledge of anxiety genetic variation, PwMS appear to have a similar genetic architecture of anxiety to that of those without MS or other immune diseases.

In two large cohorts from Canada and the UK, we found that a higher cumulative genetic burden for current anxiety symptoms was associated with increased odds for moderate anxiety symptoms in PwMS. These results were similar when stratified by sex. A previous study found a similar effect for a PGS for self‐reported anxiety in association with comorbid anxiety in a target sample of 3369 individuals with bipolar disorder (OR = 1.15, 95% CI: 1.05–1.27, *P* = 0.0034).^37^

While past studies found no association between family psychiatric history and anxiety in PwMS,^38^, ^39^ the present study provides evidence for a biological basis of anxiety symptoms in PwMS. Similarly, a genetic basis for comorbid depression in PwMS was also uncovered by our research team recently.^29^ Our methods used a direct measure of genetic variation association with current anxiety symptoms, whereas family history captures both genetic and environmental impacts.

We also found that PwMS were not at elevated odds of anxiety due to common genetic variation above that of persons without an immune disease including MS and other immune diseases. In other words, excess genetic burden did not account for the higher rates of anxiety in MS. Thus, the reason for the elevated rate of anxiety disorders in PwMS compared with the general population may be due to a multitude of factors, such as improved detection with increased healthcare use, rare genetic variation associated with anxiety, fundamentally different anxiety genetic variation specific to MS, the mental burden of a debilitating chronic neurological disease, the impact of the MS on social and physical health, or shared environmental factors such as adverse childhood experiences.^40^

The measure utilized in the present study for identifying the cumulative genetic burden associated with anxiety captures a dynamic, brief clinical process, whereas anxiety disorders are formal diagnoses. Not surprisingly, in our study, the GAD‐2 PGS was significantly associated with GAD‐2 scores and its parent symptom scale (GAD‐7). In our study, the GAD‐2 PGS was also not significantly associated with lifetime anxiety disorders as defined by either the DSM‐5 anxiety disorders criteria or with self‐reported physician‐diagnosed anxiety. This could be due to the GAD‐2 PGS capturing the genetic variability of a different construct (current anxious symptoms) rather than a lifetime formal anxiety diagnosis or other anxiety disorders such as panic disorder. This is also reflected in the recent genome‐wide association study of anxiety, whereby the genetic correlation between GAD‐2 and self‐reported physician diagnosis of an anxiety disorder was strong but not equal to 1 (*r*_*g*_ = 0.87).^13^

Our findings support further work into understanding genetic variation and PGS associated with psychiatric disorders in PwMS. They may also support the potential identification of those with newly diagnosed MS who are likely to experience moderate or greater levels of anxiety symptoms. PGS may present as a useful tool in the identification of anxiety that occurs comorbid to MS, given the overlap in somatic symptoms between anxiety and MS. Future use of PGS in screening requires further evaluation in other populations where its sensitivity and specificity can be established. The utility of PGS for this purpose is expected to improve as more genetic factors are identified. Understanding the proportion of disease risk due to genetic factors could also illuminate what proportion of disease risk may be due to modifiable factors. Identifying these modifiable factors can be facilitated by controlling for genetic differences in the risk of study participants. Although using the GAD‐2 PGS to screen for anxiety has not been assessed in clinics yet, it is theoretically possible that PGS could be a useful tool to screen subgroups where the base anxiety rate is higher than the general population, such as PwMS. Based on a recent systematic review, the US Preventative Services Task Force recommended screening for anxiety disorders in adults.^41^ Those PwMS at higher risk for anxiety may be offered preventative strategies, such as cognitive behavioral therapy, to strengthen resilience. In children with a family history of an anxiety disorder, none of those completing an 8‐week family‐based cognitive behavioral therapy intervention developed anxiety compared with one‐third of those who did not.^42^

Our study had many strengths, including a large, multi‐country sample with multiple measures that examined both lifetime history and current levels of anxiety, which helped to establish the generalizability and replication of our findings. However, our study also had its limitations. We included the UKB in our study due to its size and extensiveness, however, there is a known healthy volunteer bias in the UKB (i.e., those in UKB are, on average, “healthier”),^43^ which likely led to the lower anxiety rates compared to our MS clinic cohorts from Canada and the USA and was apparent from the effect size for self‐reported anxiety in the UKB. We attempted to balance the limitations of the UKB with its advantages, including its size and extensive phenotype measures,^44^ while including other cohorts that supplemented our cohort of PwMS. Another limitation relates to the heterogeneous nature of anxiety disorders and our lack of anxiety subtype information or in the case of the US cohort which lacked a structured anxiety measure.

Our findings highlight that future studies deciphering the genetic heterogeneity of anxiety in PwMS will be useful and further understanding of the pathophysiology of anxiety in PwMS may benefit from investigating the subtypes of anxiety disorders, including panic disorders. Further to this, investigating MS‐specific anxiety traits could also assist with unraveling the heterogeneity of comorbid anxiety in MS, for which our sample size may have been limited to identify. Our findings also suggest that future studies investigating the etiology of anxiety in MS should control for genetic factors. Our results were also only generalizable to PwMS of European genetic ancestry as we excluded the small number of non‐European genetic ancestry samples from PwMS to ensure high transferability from the original GAD‐2 genome‐wide association study to maximize PGS association in our target samples.^13^, ^30^ There is a need for targeted recruitment of non‐European ancestry participants with MS^45^, ^46^ and collection of extensive phenotype information for future studies and determining the applicability of PGS between ancestries. We were unable to exclude PwMS from the previous GAD‐2 genome‐wide association study,^13^ however, a previous study using a larger version of the cohort used in the genome‐wide study reported the number of individuals with MS to be low (0.8%).^47^

Collectively, we observed that in PwMS of European genetic ancestry, for each standard deviation increase in the GAD‐2 PGS, there was a ~50% increase in the occurrence of moderate anxiety symptoms. This effect did not differ by sex and whether the anxiety symptoms occurred as a comorbid symptom of MS or in individuals with no immune disease. Future studies assessing these findings in other European ancestry MS cohorts and in individuals of non‐European genetic ancestry will be important to establish the generalizability of this finding. In conclusion, our findings indicate a genetic basis of anxiety in PwMS which is like that seen in individuals with no immune disease.

## Author Contributions

KK, FDL, and RAM: conception and design of the study, acquisition and analysis of data, and drafting a significant portion of the manuscript or figures; AH: acquisition and analysis of data and 3) drafting a significant portion of the manuscript or figures; CD, SH, and AP: acquisition and analysis of data; KCF, AS, CNB, JMB, GC, JDF, JG, LAG, CAH, DFL, SP, MBS, and HKT: conception and design of the study, acquisition and analysis of data; YL, KAM, and JSW: conception and design of the study.

## Conflict of Interest

**Dr. Salter** receives research funding from the Multiple Sclerosis Society of Canada, the National Multiple Sclerosis Society, CMSC, and the US Department of Defense and is a member of the editorial board for Neurology. She serves as a consultant for Gryphon Bio, LLC. She is a member of the Data and Safety Monitoring Board for Premature Infants Receiving Milking or Delayed Cord Clamping (PREMOD2), Central Vein Sign: A Diagnostic Biomarker in Multiple Sclerosis (CAVS‐MS), Ocrelizumab for Preventing Clinical Multiple Sclerosis in Individuals with Radiologically Isolated Disease (CELLO) and Methotrexate treatment of Arthritis caused by Chikungunya virus (MARCH). **Dr. Bernstein** is supported by the Bingham Chair in Gastroenterology. He has served on advisory Boards for AbbVie Canada, Amgen Canada, Bristol Myers Squibb Canada, Eli Lilly Canada, Ferring Canada, JAMP Pharmaceuticals, Janssen Canada, Pendopharm, Sandoz Canada, Takeda Canada, and Pfizer Canada; Consultant for Mylan Pharmaceuticals and Takeda; Received educational grants from Abbvie Canada, Amgen Canada, Bristol Myers Squibb Canada, Eli Lilly Canada, Ferring Canada, Pfizer Canada, Takeda Canada, and Janssen Canada. Received research funding from Abbvie Canada, Amgen Canada, Pfizer Canada, Sandoz Canada, and Takeda Canada. **Dr. Fisk** receives research grant support from the Canadian Institutes of Health Research, the National Multiple Sclerosis Society, MS Canada, Crohn's and Colitis Canada, Research Nova Scotia; and consultation and distribution royalties from MAPI Research Trust. **Dr. Graff** has received research funding from Pfizer Canada and Takeda Canada. **Dr. Hitchon** has served on advisory boards for Astra‐Zeneca and received research funding from Pfizer. **Dr. Gelernter** is named as an inventor on PCT patent application #15/878,640 entitled: “Genotype‐guided dosing of opioid agonists,” filed on January 24, 2018, and issued on January 26, 2021 as U.S. Patent No. 10,900,082. Dr. Gelernter is paid for editorial work for the journal Complex Psychiatry. **Dr. Cutter** has the following disclosures: Data and Safety Monitoring Boards for Applied Therapeutics, AI therapeutics, AMO Pharma, Astra‐Zeneca, Avexis Pharmaceuticals, Bristol Meyers Squibb/Celgene, CSL Behring, Cynata Therapeutics, Horizon Pharmaceuticals, Immunic, Karuna Therapeutics, Kezar Life Sciences, Mapi Pharmaceuticals LTD, Merck, Mitsubishi Tanabe Pharma Holdings, Opko Biologics, Prothena Biosciences, Novartis, Regeneron, Sanofi‐Aventis, Reata Pharmaceuticals, Teva Pharmaceuticals, NHLBI (Protocol Review Committee), University of Texas Southwestern, University of Pennsylvania, Visioneering Technologies, Inc; and Consulting or Advisory Boards for Alexion, Antisense Therapeutics, Avotres, Biogen, Clene Nanomedicine, Clinical Trial Solutions LLC, Endra Life Sciences, Entelexo Biotherapeutics, Inc., Genzyme, Genentech, GW Pharmaceuticals, Hoya Corporation, Immunic, Immunosis Pty Ltd, Klein‐Buendel Incorporated, Linical, Merck/Serono, Novartis, Perception Neurosciences, Protalix Biotherapeutics, Regeneron, Roche, SAB Biotherapeutics. Dr. Cutter is employed by the University of Alabama at Birmingham and President of Pythagoras, Inc. a private consulting company located in Birmingham, AL. **Dr. K McKay** receives research funding from StratNeuro and has received speaker honoraria from Biogen and Sanofi. **Dr. MB Stein** has stock options in Oxeia Biopharmaceuticals and EpiVario. He has been paid for his editorial work on Depression and Anxiety (Editor‐in‐Chief), Biological Psychiatry (Deputy Editor), and UpToDate (Co‐Editor‐in‐Chief for Psychiatry). He has also received research support from NIH, Department of Veterans Affairs, and the Department of Defense. He is on the scientific advisory board for the Brain and Behavior Research Foundation and the Anxiety and Depression Association of America. **Dr. J. Wolinsky** received compensation for consulting, scientific advisory boards, or other activities with the Cleveland Clinic Foundation, EMD Serono, Inmagene, Novartis, Roche/Genentech, Sandoz, University of Alabama, and Zenas BioPharm. Royalties are received for our licensed monoclonal antibodies through UTHealth to Millipore (Chemicon International) Corporation. Dr Kowalec, Mr. Harder, Ms. Dolovich, Dr. KC Fitzgerald, Dr. Y Lu, Dr. JM Bolton, Dr. S Hägg, Dr. DF Levey, Dr. FD Lublin, Dr. S Patten, Dr. A Patki, Dr HK Tiwari, and Dr. RA Marrie report no disclosures related to this work.

## Supporting information


**Data S1.** Supplementary methods.
**Figure S1.** Overlap in the three categorical definitions of anxiety used in the (A, B) Canadian and (C, D) UK Biobank (UKB) samples.
**Table S1.** List of conditions and corresponding ICD‐10 codes and survey question number for use in defining healthy controls in UKB and Canada samples.
**Table S2.** Variance explained in anxiety outcome by the GAD‐2 polygenic score as measured by Nagelkerke pseudo‐*R* ^2^, expressed as a percentage.
**Table S3.** Number of participants included in the meta‐analyses.
**Table S4.** Multivariable logistic regression analyses investigating the association between the GAD‐2 polygenic score with multiple sclerosis (MS) and comorbid anxiety and (A) anxiety and no comorbid immune disease and (B) healthy controls.
**Table S5.** Number of females and males included in sex‐stratified regression of MS/anxiety compared to MS/no anxiety, as defined by three categorical anxiety measures.
**Table S6.** Sex‐stratified multivariable logistic regression investigating the association between the polygenic score for GAD‐2 and comorbid anxiety in multiple sclerosis.

## Data Availability

Individual data collected for the USA cohort cannot be shared but access may be granted via authorization from the CombiRx Trial Executive Committee. Some Canadian participants did not consent to their data being shared outside the original study; thus, for those participants who did consent to their data being shared outside the original study, reasonable requests for access to their data can be made to Professor Ruth Ann Marrie with the appropriate ethical approvals and data sharing agreements. An application to access UK data is available via the UK Biobank. Genome‐wide association study summary statistics for the GAD‐2 PGS computation were made available through the Million Veterans Project (Approved Project MVP025: Genomics of Posttraumatic Stress Disorder, PI: Stein and Gelernter). The Analyses code is available on GitHub (https://github.com/kkowalec/ms‐anxiety).
