# Supplementary material for: Polygenic liability for anxiety in association with comorbid anxiety in multiple sclerosis
Source: Ann Clin Transl Neurol. 2024 May 7;11(6):1393–404. doi: 10.1002/acn3.52025 (PMC11187942; doi:10.1002/acn3.52025)
Supplement: Supplementary file 1 — Data S1. Supplementary methods. Figure S1. Overlap in the three categorical definitions of anxiety used in the (A, B) Canadian and (C, D) UK Biobank (UKB) samples. Table S1. List of conditions and corresponding ICD‐10 codes and survey question number for use in defining healthy controls in UKB and Canada samples. Table S2. Variance explained in anxiety outcome by the GAD‐2 polygenic score as measured by Nagelkerke pseudo‐R 2, expressed as a percentage. Table S3. Number of participants included in the meta‐analyses. Table S4. Multivariable logistic regression analyses investigating the association between the GAD‐2 polygenic score with multiple sclerosis (MS) and comorbid anxiety and (A) anxiety and no comorbid immune disease and (B) healthy controls. Table S5. Number of females and males included in sex‐stratified regression of MS/anxiety compared to MS/no anxiety, as defined by three categorical anxiety measures. Table S6. Sex‐stratified multivariable logistic regression investigating the association between the polygenic score for GAD‐2 and comorbid anxiety in multiple sclerosis. [file ACN3-11-1393-s001.docx]

**Supplementary Methods**

**Study samples**

1. **Canada/IMID study**

Multiple recruitment methods were used such as advertisements placed in private medical clinics, hospitals, and educational institutions. For the PwMS, recruitment included in-person contact, telephone calls or mailouts from community-based and tertiary care clinics. Blood samples were collected in addition to extensive phenotypes. Participants had to be sufficiently proficient in English to complete study questionnaires and were ≥18 years old.

1. **United Kingdom/UK Biobank**

The UK Biobank (UKB) is a population-based cohort of ~500,000 individuals aged 37-73 years from the UK, recruited from 2006 through 2010.^1^ At an assessment center, participants answered touchscreen questionnaires about diseases, which was then followed by a research nurse-led interview for further details regarding the diseases reported. Linked hospital records using international classification of diseases (ICD)-version 10 codes were also utilized to identify diseases. In addition, ~158,000 individuals from the original cohort completed a web-based mental health questionnaire, which included the Composite International Diagnostic Interview-Short Form (CIDI-SF).^2^ Blood samples were collected. From this cohort, we selected PwMS, individuals with a lifetime history of anxiety disorders (no immune disease), and healthy controls.

1. **USA/CombiRx**

The CombiRx trial was a randomized, multi-center, Phase-III clinical trial of combination MS disease-modifying therapies (interferon-β1a and glatiramer acetate vs. either agent alone; ClinicalTrial.gov: NCT00211887).^3^ Study criteria included neurologist-confirmed MS, with ≥2 relapses in the previous 3 years; aged 18-60 years, an EDSS of 0-6 and no relapses in the 30 days prior to screening and randomization. Completion of the 3-year core study was then extended to include an additional 4 years (7 years total follow-up). Blood samples were collected. These samples were utilized only for comparing comorbid anxiety in PwMS to MS without anxiety, given this was a clinical trial sample of only PwMS.

**Participant definitions**

We included one case group and three control groups: (1) PwMS and anxiety (cases), (2) PwMS and no anxiety (control), (3) anxiety and no immune disease (control), and (4) healthy (control).

Of particular importance in genetic studies, we included data from three different studies and countries to improve precision of our estimates. The anxiety phenotypes were harmonized between the studies, which included comparing a gold standard measure of anxiety disorders (Structured Clinical Interview for the Diagnostic and Statistical Manual of Mental Disorders, SCID-DSM-5) in the Canadian sample with that of a comparable version in the UKB sample combining ICD-10 diagnoses and the CIDI-SF. We then expanded this to include a self-reported measure of anxiety and another related, but different construct: current anxiety symptoms (GAD-7) that was measured in both the Canadian and UKB cohorts. The USA sample had only one measure of anxiety, which was self-reported anxiety.

**UKB measures**

Self-report questionnaires captured the following at the baseline visit: age, sex, BMI (underweight: <18.5, healthy: 18.5-24.9, overweight: 25-29.9, obese: ≥30.0 ^4^), annual household income (converted from British Pounds [GBP] to Canadian Dollars [CAD] 1 GBP=1.61 CAD, and into: <$50,000, ≥$50,000, or “declined to answer”), highest education ([high school or lower]: A levels/AS levels or equivalent, Certificate of Secondary Education or equivalent, O levels/General Certificate of Secondary Education or equivalent vs. [Above high school]: National Vocational Qualification or Higher National Diploma or higher National Certificate or equivalent, College or University degree, Other professional qualifications e.g.: nursing, teaching vs. [Other]), and years of education. Smokers were defined as ever if reporting current or ever tobacco smoking on most days or occasionally.

**Genotyping, quality control, and imputation**

Genotype data was available for 409 Canadian, 599 USA, and 487,410 UK participants following central quality control and imputation. Canadian samples were genotyped on the Illumina Global Screening Array SNP (v2) and were subject to quality control and imputed using the Michigan Imputation Service (Haplotype Reference Consortium, r1.1).^5,6^ UK samples were genotyped using the Affymetrix UK Biobank Axiom Array, with full details reported previously.^1^  and imputed with the Haplotype Reference Consortium (r1.1) panel. USA samples were genotyped using HumanOmni1-Quad chip array at the Translational Genomics Research Institute, Arizona and imputed using TOPMed reference panel. To determine genetic ancestry, we performed principal components analysis in PLINK using the 1000 Genomes phase 3 v5 data as the reference (N=2,493 unrelated individuals, by “Superpopulation”: 659 African, 347 Admixed, 504 East Asian, 503 Europeans, 480 South Asian) along with each cohorts study data.^7^ We excluded any samples that were further than three standard deviations from the 1000 Genomes European superpopulation reference on principal components 1 or 2 (N removed: 75 Canada; 27 700 UKB; 21 USA).^7^ Upon removing the non-European genetic ancestry participants, the principal components were regenerated without the reference data and were used as covariates for each individual cohort. From all three cohorts, genotyped and imputed variants were subject to site-specific quality control and all analyses were run in each cohort separately and then meta-analyzed.

**PGS generation**

Polygenic scores were calculated using summary statistics from recent genome-wide association studies for each phenotype: GAD-2.^8^ PGS were generated as the sum of the risk allele scores, weighted by the effect size from the GAD-2 genome-wide association study.^8^ In the Canadian and USA samples, we performed linkage disequilibrium clumping (r^2^<0.1 in 1-Mb window) on the overlapping genetic variants with the 1000 Genomes Project European samples for the reference. Using PLINK (v1.9), PGS were calculated at 8 different p-value thresholds (≤5×10^−8^, ≤1×10^−5^, ≤1×10^−3^, ≤0.01, ≤0.05, ≤0.1, ≤0.5, and ≤1). We computed the variance in outcome [Nagelkerke’s pseudo-*R^2^* ^9^] explained by the GAD-2 PGS as the difference in R^2^ from a logistic regression model including the GAD-2 PGS and a baseline model including only genetic ancestry principal components (***Table S2***) to determine which p-value threshold to include in our final modelling. For the UKB sample, PGS were computed using the SBayesR module in GCTB,^10^ and PLINK (v1.9). We standardized all PGS to a mean of 0 (standard deviation=1) for ease of interpretation.

**eFigure 1** Overlap in the three categorical definitions of anxiety used in the (A-B) Canadian and (C-D) UK Biobank (UKB) samples.

**
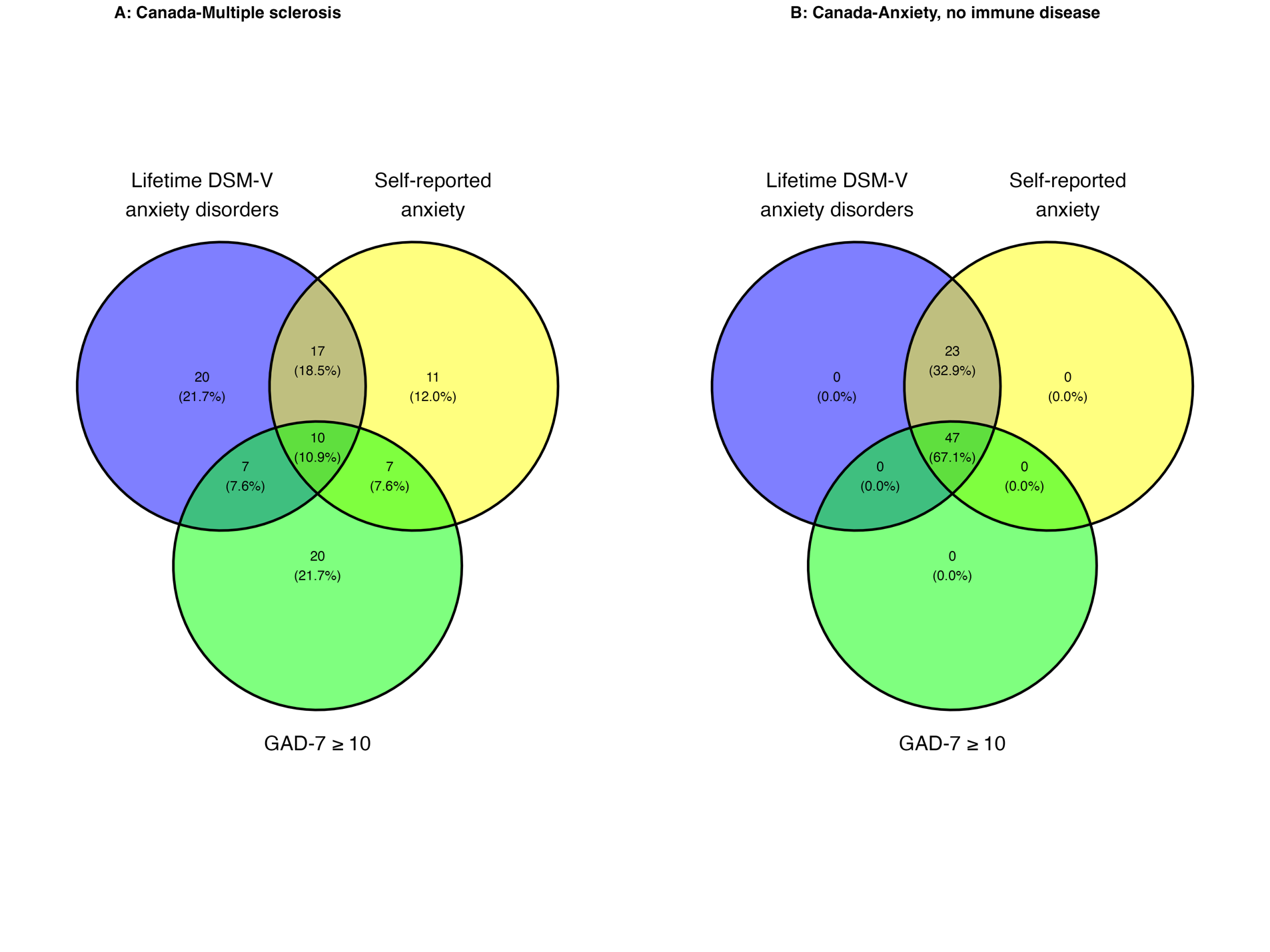
**

**
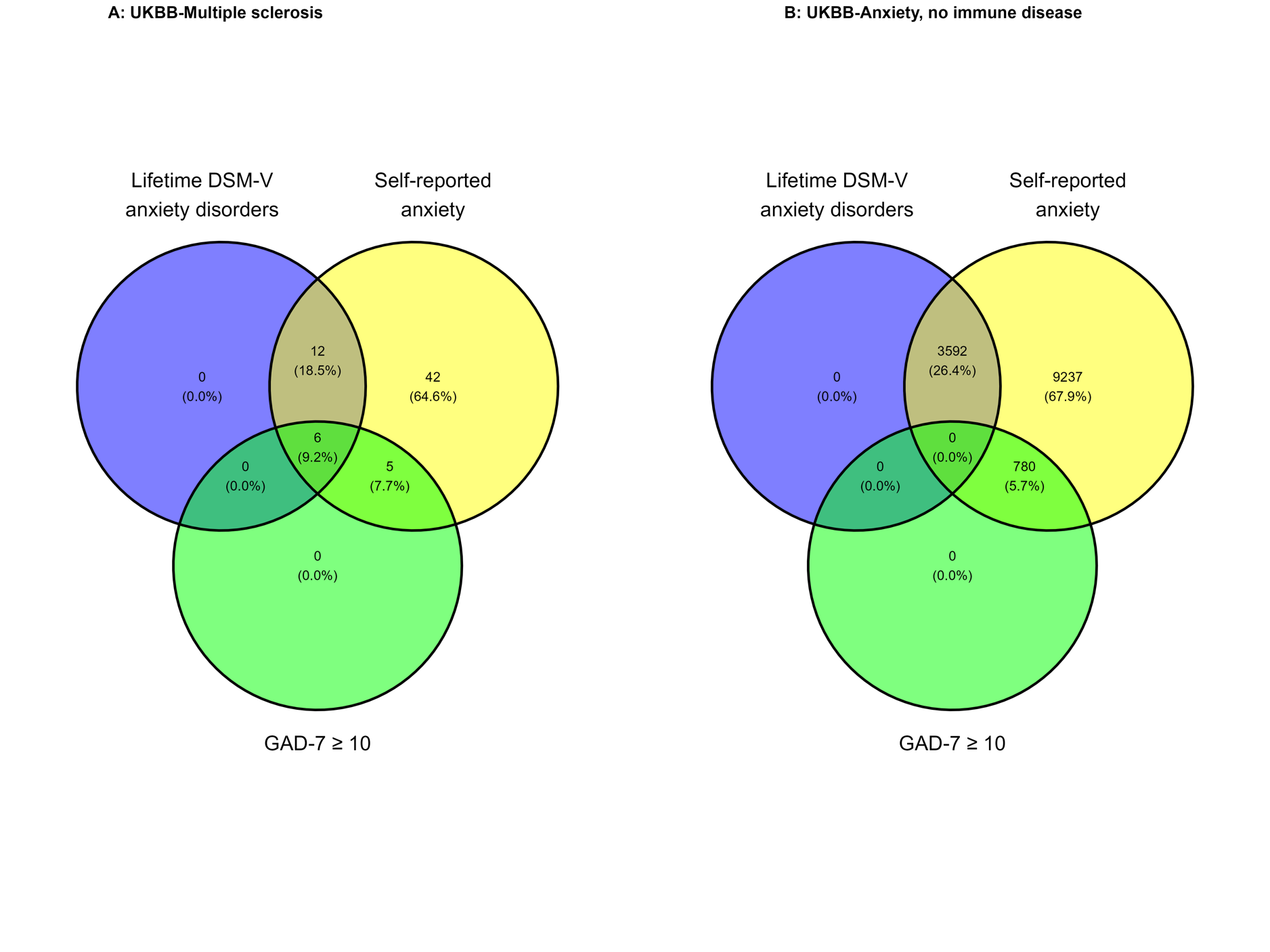
**

**Table S1:** List of conditions and corresponding ICD-10 codes and survey question number for use in defining healthy controls in UKB and Canada samples.

| Condition | ICD-10 code (UKB) | Baseline survey data fields (UKB) |
| --- | --- | --- |
| High cholesterol (hyperlipidemia) | E780, E782, E784, E785^11^ | 1473 |
| High blood pressure (hypertension) | I10–I13, I15^11^ | 1065, 1072 |
| Heart trouble (such as angina, congestive heart failure, or coronary artery disease, myocardial infarction) | I20–I25^11^ | 1066, 1074 |
| Disease of arteries in the legs (Peripheral vascular disease) | I70, I73.8, I73.9^12^ | 1067, 1087, 1088 |
| Asthma, emphysema, chronic bronchitis, or chronic obstructive pulmonary disease | J45, J46, J43, J40-J42, J42 | 1111, 1472, 1113, 1112 |
| Diabetes mellitus (type 1/type 2) | E10, E11 | 1220^13^; 1222 (type 1), 1223 (type 2) |
| Cancer of the breast/colon/lung/skin or others | C0-C33, C34, C45-C49, C51-C60, C62-C97 | 2453 |
| Migraine | G43 | 1265^14^ |
| Thyroid disease | E2, E3, E05, E06 | 1226, 1225, 1428 |
| Lupus (systemic lupus erythematosus) | M32 | 1381 |
| Degenerative arthritis (osteoarthritis) | M15, M16, M17, M18, M19, M47 | 1465^15^ |
| Osteoporosis | M80, M81, M82 | 1309 |
| Fibromyalgia | M79.7 | 1542 |
| Kidney disease | N10-N19^16^ | 1192, 1193, 1194, 1405 |
| Peptic ulcer disease, gastroesophageal reflux disease (GERD) | K25 (peptic ulcer)  K21 (GERD) | 1400, 1138 |
| Liver problems (e.g. cirrhosis, Hepatitis B, Hepatitis C, Fatty Liver) | K70-K77 | 1158, 1579, 1580 |
| Irritable bowel syndrome | K58 | 1154 |
| Epilepsy (seizure disorder) | G40, G41^11^ | 1264 |
| Depression | F32–F34^11^ | 1286 |
| Anxiety disorder | F40, F41^11^ | 1287 |
| Bipolar disorder | F31^17^ | 1291 |
| Schizophrenia | F20 | 1289 |
| Other psychiatric disorders (substance use, ADHD, eating disorders, personality disorders) | F10-F19 (-F17), K70, F50, F60, F90 | 1408, 1409, 1410, 1604, 1470 |
| Immune diseases: inflammatory bowel disease, multiple sclerosis, rheumatoid arthritis, celiac, psoriasis, Sjogren’s | K50, K51^11^, G35, M5, M6, K90, L40, M35 | 1461, 1261, 1464, 1456, 1453, 1382 |
| Brain injury (concussions or other trauma) | S02, S06, S09^18^ | 1266 |
| Polycystic ovarian syndrome | E28 | 1350 |

This list of conditions is those that were excluded to define the Canadian healthy control group using self-reported questionnaires. In addition to define the control group with anxiety and no immune disease, we used the list of immune diseases here.

**Table S2:** Variance explained in anxiety outcome by the GAD-2 polygenic score as measured by Nagelkerke pseudo-R^2^, expressed as a percentage.

| P-value threshold or method | Canada | UK | USA |
| --- | --- | --- | --- |
| p≤5e-8 | 0.08 | N/A | 0.00 |
| p≤1e-5 | 0.03 | N/A | 0.80 |
| p≤1e-3 | 1.25* | N/A | 0.07 |
| p≤0.01 | 0.14 | N/A | 1.03* |
| p≤0.05 | 0.00 | N/A | 0.46 |
| p≤0.1 | 0.40 | N/A | 0.68 |
| p≤0.5 | 0.45 | N/A | 0.14 |
| p≤1 | 0.32 | N/A | 0.10 |
| SBayesR | N/A | 1.08* | N/A |

Outcome used: GAD-2 (Canada and UK), self-reported anxiety in multiple sclerosis (USA). R^2^ expressed as a percentage (%). *Represents the selected p-value threshold for analyses.

**Table S3:** Number of participants included in the meta-analyses.

| Comparison^A^ | | | | | | | | | |  |
| --- | --- | --- | --- | --- | --- | --- | --- | --- | --- | --- |
|  | 1. Multiple sclerosis-anxiety (case) compared to multiple sclerosis-no anxiety (control) | | | 1. Multiple sclerosis-anxiety (case) compared to anxiety-no immune disease (control) | | | 1. Multiple sclerosis-anxiety (case) compared to healthy (control) | | |  |
|  | **Total** | **Case** | **Control** | **Total** | **Case** | **Control** | **Total** | **Case** | **Control** | |
| Outcome |  |  |  |  |  |  |  |  |  | |
| Current GAD-7≥10 | 571 | 70 | 501 | 3,134 | 70 | 3,064 | 12,485 | 70 | 12,415 | |
| Lifetime DSM-5 anxiety disorders | 1,552 | 101 | 1,451 | 12,626 | 101 | 12,525 | 54,368 | 101 | 54,267 | |
| Lifetime self-reported physician diagnosed anxiety | 2,119 | 229 | 1,890 | 15,006 | 110 | 14,896 | 54,377 | 110 | 54,267 | |

^A^ Comparisons are as follows: 1) Canada, UK for all three outcomes and Canada, USA, UK for self-reported anxiety. 2) and 3) are for Canada and UK only as the specified control groups [anxiety no immune disease and healthy controls] were not recruited in the USA sample as it is from a MS clinical trial.

**Table S4:** Multivariable logistic regression analyses investigating the association between the GAD-2 polygenic score with multiple sclerosis (MS) and comorbid anxiety and (A) anxiety and no comorbid immune disease and (B) healthy controls.

|  | A: MS-anxiety (case) compared to  anxiety-no immune disease (control) | | | B: MS-anxiety (case) compared to  healthy controls | | |
| --- | --- | --- | --- | --- | --- | --- |
|  | **Canada** | **UKB** | **Meta-Analysis** | **Canada** | **UKB** | **Meta-Analysis** |
| Binary outcome |  |  |  |  |  |  |
| Current GAD-7≥10 | 0.62 (0.35-1.06), 0.08 | 1.33 (0.89-1.97), 0.5 | 1.02 (0.74-1.40),  0.9, 80.5%^a^ | 0.93 (0.55-1.58), 0.8 | **1.85 (1.25-2.75), 0.002** | **1.45 (1.05-1.98), 0.02, 75.8%** ^a^ |
| Lifetime DSM-5 anxiety disorders | 1.02 (0.99-1.05), 0.6 | 0.95 (0.72-1.27), 0.7 | 0.94 (0.75-1.19),  0.6, 0% | 1.03 (0.63-1.72), 0.9 | 1.12 (0.83-1.48), 0.5 | 1.10 (0.85-1.41), 0.5, 0% |
| Lifetime self-reported physician diagnosed anxiety | 0.71 (0.45-1.05), 0.09 | 0.93 (0.73-1.19), 0.6 | 0.87 (0.71-1.08),  0.2, 32.1% | 0.80 (0.50-1.29), 0.3 | 1.02 (0.80-1.30), 0.8 | 0.97 (0.78-1.21), 0.8, 0% |

Each outcome is assessed as a separate model includes the polygenic score for anxiety, the first 5 genetic ancestry principal components, age, and sex. Data represented as: odds ratio, (95%CI), P-value, I^2^ (for meta-analyses). Bolded p-value indicates P≤0.05. ^a^Random-effect inverse-variance weighted model, whereas others used a fixed-effect.

**Table S5:** Number of females and males included in sex-stratified regression of MS-anxiety compared to MS-no anxiety, as defined by three categorical anxiety measures.

|  | Canada  (N=213 MS) | | UKB  (N=358-1390 MS) ^A^ | | USA  (N=578 MS) | | Meta-analyses | |
| --- | --- | --- | --- | --- | --- | --- | --- | --- |
| Binary outcome | **Females** | **Males** | **Females** | **Males** | **Females** | **Males** | **Females** | **Males** |
| Current GAD-7≥10 | 36 cases,  138 controls | 8 cases,  31 controls | 22 cases,  256 controls | 4 cases,  76 controls | N/A | N/A | 58 cases, 394 controls | 12 cases, 107 controls |
| Lifetime DSM-5 anxiety disorders | 50 cases,  124 controls | 4 cases,  45 controls | 39 cases,  935 controls | 8 cases,  357 controls | N/A | N/A | 139 cases, 1,059 controls | 12 cases, 402 controls |
| Lifetime self-reported physician diagnosed anxiety | 41 cases,  132 controls | 4 cases,  45 controls | 52 cases,  914 controls | 13 cases,  350 controls | 93 cases,  324 controls | 26 cases,  135 controls | 186 cases, 1,370 controls | 43 cases, 530 controls |

A: For UKB, the denominator for the DSM-5 anxiety disorders and self-reported anxiety is N=1,390 multiple sclerosis cases. For the GAD-7 ≥10 outcome, the denominator is N=358 multiple sclerosis cases as these were the subset of UKB with an additional mental health questionnaire.

**Table S6:** Sex-stratified multivariable logistic regression investigating the association between the polygenic score for GAD-2 and comorbid anxiety in multiple sclerosis.

|  | Canada | | UK | | USA | | Meta-Analysis | |
| --- | --- | --- | --- | --- | --- | --- | --- | --- |
| Binary outcome | **Female** | **Male** | **Female** | **Male** | **Female** | **Male** | **Female** | **Male** |
| Current GAD-7≥10 | 1.16 (0.77-1.76), 0.4 | 3.9 (0.6-2.5), 0.1 | **1.99 (1.16-3.39), 0.01** | 3.84 (0.43-34.3), 0.2 | N/A | N/A | **1.43 (1.03-1.97), 0.03, 58.8%** ^a^ | 3.89 (0.94-16.1), 0.06, 0% |
| Lifetime DSM-5 anxiety disorders | 1.28 (0.89-1.83), 0.2 | 3.78 (0.62-2.2), 0.1 | 0.95 (0.67-1.32), 0.7 | 1.73 (0.81-3.66), 0.1 | N/A | N/A | 1.09 (0.85-1.39), 0.52, 24.6% | 1.94 (0.98-3.86), 0.06, 0% |
| Lifetime self-reported physician diagnosed anxiety | 0.93 (0.64-1.35), 0.7 | 2.20 (0.36-13.2), 0.3 | 0.92 (0.69-1.23), 0.6 | 0.95 (0.52-1.76), 0.8 | 1.21 (0.95-1.53), 0.2 | 1.14 (0.75-1.7), 0.5 | 0.99 (0.81-1.20), 0.89, 0% | 1.13 (0.90-1.41), 0.28, 0% |

Data represented as: odds ratio (95%CI); P-value for binary outcomes, and I^2^ (for meta-analyses). Bolded p-value indicates P≤0.05. ^a^Random-effect inverse-variance weighted model, whereas others used a fixed-effect.

The outcome is multiple sclerosis-anxiety (case) compared to multiple sclerosis-no anxiety (control) in females or males. Each anxiety measure is assessed as a separate model and includes the GAD-2polygenic score, the first 5 genetic ancestry principal components, and age.

In the unstratified models (Table 2), we included a sex*GAD-2 PGS interaction term but none were significant: Canada: DSM-5 ß=-0.62, P=0.36, self-reported anxiety ß=-0.71, P=0.29, GAD-7≥10 ß=-0.37, P=0.52; USA: self-reported anxiety ß=0.002, P=0.99; UKB: DSM-5 ß=-0.46, P=0.25, self-reported anxiety ß=2e-5, P=0.99, GAD-7≥10 ß=0.35, P=0.65.
